# Sodium Alginate as a Potential Therapeutic Filler: An In Vivo Study in Rats

**DOI:** 10.3390/md18100520

**Published:** 2020-10-19

**Authors:** Masanori Mori, Rintaro Asahi, Yoshihiro Yamamoto, Takanobu Mashiko, Kayo Yoshizumi, Natsumi Saito, Takako Shirado, Yunyan Wu, Kotaro Yoshimura

**Affiliations:** Department of Plastic Surgery, Jichi Medical University, Tochigi 329-0498, Japan; mamori-kyt@umin.ac.jp (M.M.); asahi-rntr@umin.ac.jp (R.A.); yoshihiro_yama@jichi.ac.jp (Y.Y.); Takanobu-mashiko@umin.ac.jp (T.M.); kyosizumi-ama@umin.ac.jp (K.Y.); natsaito@jichi.ac.jp (N.S.); shirado@jichi.ac.jp (T.S.); wu-yunyan@jichi.ac.jp (Y.W.)

**Keywords:** filler, sodium alginate, alginic acid, sodium hyaluronate, hyaluronic acid, granuloma, capsule

## Abstract

Filler injection demand is increasing worldwide, but no ideal filler with safety and longevity currently exists. Sodium alginate (SA) is the sodium salt of alginic acid, which is a polymeric polysaccharide obtained by linear polymerization of two types of uronic acid, d-mannuronic acid (M) and l-guluronic acid (G). This study aimed to evaluate the therapeutic value of SA. Nine SA types with different M/G ratios and viscosities were tested and compared with a commercially available sodium hyaluronate (SH) filler. Three injection modes (onto the periosteum, intradermally, or subcutaneously) were used in six rats for each substance, and the animals were sacrificed at 4 or 24 weeks. Changes in the diameter and volume were measured macroscopically and by computed tomography, and histopathological evaluations were performed. SA with a low M/G ratio generally maintained skin uplift. The bulge gradually decreased over time but slightly increased at 4 weeks in some samples. No capsule formation was observed around SA. However, granulomatous reactions, including macrophage recruitment, were observed 4 weeks after SA implantation, although fewer macrophages and granulomatous reactions were observed at 24 weeks. The long-term volumizing effects and degree of granulomatous reactions differed depending on the M/G ratio and viscosity. By contrast, SH showed capsule formation but with minimal granulomatous reactions. The beneficial and adverse effects of SA as a filler differed according to the viscosity or M/G ratio, suggesting a better long-term volumizing effect than SH with relatively low immunogenicity

## 1. Introduction

Many types of fillers are now widely used for cosmetic and reconstructive purposes but there is no ideal filler yet [1]. An ideal filler should be effective in shaping and volumizing, long-lasting, biocompatible, safe, and low in cost. Hyaluronic acid (sodium hyaluronate, SH), which is currently the most widely used filler, is effective and safe [2], but it is also expensive per volume and not long-lasting [3].

Sodium alginate (SA) is a highly biocompatible substance [4] that is now used in a wide range of fields, from food additives [5] to pharmaceuticals [6]. SA is the sodium salt of alginic acid extracted from brown algae [7], and is inexpensive compared with other medical materials. In recent years, research has been conducted on the use of SA as a bioengineering scaffold for tissue regeneration in the field of regenerative medicine [8]. SA also has mucosal-protective and hemostatic effects [9,10], and two approved SA pharmaceuticals exist in Japan. One is an oral solution of SA (Alloid G; Kaigen Pharma) to treat gastroduodenal ulcer and hemostasis after gastric biopsy. The other is a temporal injectable filler (Liftal; Kaigen Pharma, Osaka, Japan), which is injected into the base of the gastric polyp immediately before endoscopic polypectomy to facilitate the polypectomy procedure [11].

Alginic acid is a polymeric polysaccharide obtained by the linear polymerization of two uronic acid types, d-mannuronic acid and l-guluronic acid [12]. The quantitative ratio of d-mannuronic acid and l-guluronic acid (M/G ratio) differs depending on the origin (type of brown algae) [13]. The viscosity of SA solution depends on the degree of polymerization (the higher the degree of polymerization, the higher the viscosity) and can be manipulated by heating or enzymatic treatment [14]. The M/G ratio has been reported to affect macrophage activity [15] and viscosity may greatly also influence the property of SA as a filler. In this study, we tested nine types of 4% SA phosphate-buffered saline solutions with different M/G ratios or viscosities, which were referred to as SA1 to SA9, in four-week and 24-week rat models. These nine types are combinations of high, medium, and low M/G ratio with high, medium, and low viscosity, respectively. We also used a commercial sodium hyaluronate (SH) as the control substance. This study aimed to evaluate the potential of different types of SA solutions as therapeutic fillers.

## 2. Results

### 2.1. Macroscopic Findings

Infection was observed in one site with the injection of SA5 at 4 weeks, and no other infection was detected among the 300 injected sites in 60 rats. No other changes were noted in the other injection sites, such as inflammatory signs, other than bulging. No macroscopic change was found in any of the phosphate-buffered saline (PBS)-injected sites.

**Figure 1:**
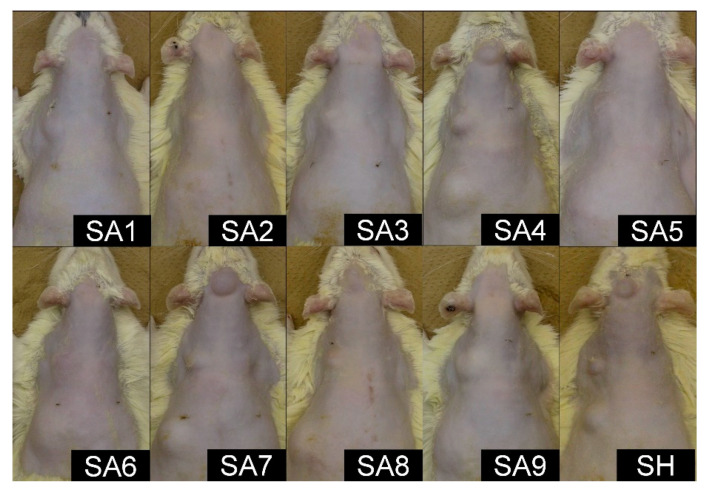
Representative macroscopic views at 24 weeks. Some types of sodium alginate were almost flattened, but other types, such as SA4, SA7, and SA8, as well as SH, maintained a projected shape. SA: sodium alginate, SH, sodium hyaluronate.

At 24 weeks, the substances injected intradermally were macroscopically detected in the skin of 10 rats (one in SA1, one in SA2, one in SA3, two in SA4, one in SA5, two in SA6, and two in SA7) (Figure 1). However, almost all the rats with intradermal injection of SA showed a skin bulge due to the subcutaneous substances, suggesting that a major proportion of the substance injected intradermally was leaked into the subcutis. No changes were observed in the other injection sites except for bulging. No macroscopic change was observed in any of the injection sites with PBS.

### 2.2. Sequential Changes in the Diameter and Volume

All the results of the diameters are shown in Table S1. The diameters at day 0 were measured immediately after injection. Because all the SA and SH types absorbed water and horizontally expanded after injection, the diameters at 4 weeks were larger than those at day 0 in all the experimental groups. After 4 weeks, the diameters declined gradually. Some bulges in the periosteum and intradermal groups became so flattened that the diameters could not be measured. All the injected sites with PBS were completely flat at 4 weeks.

All the results of the volume are shown in Table S2. The volumes at day 0 were measured by computed tomography (CT) scanning about 1 h after injection. The volumes of SA at day 0 were 2–3 times as large as the injected volumes, whereas the expansion of SH was within 1.5 times. The volumes of the injected SA and SH decreased at 4 weeks, and the decrease in volume was slower after 4 weeks. At 8 weeks, some groups showed a subtle increase in volume. By contrast, the volume could not be measured in the other flattened groups because the boundary around the injected substances was unclear in the CT images.

**Figure 2:**
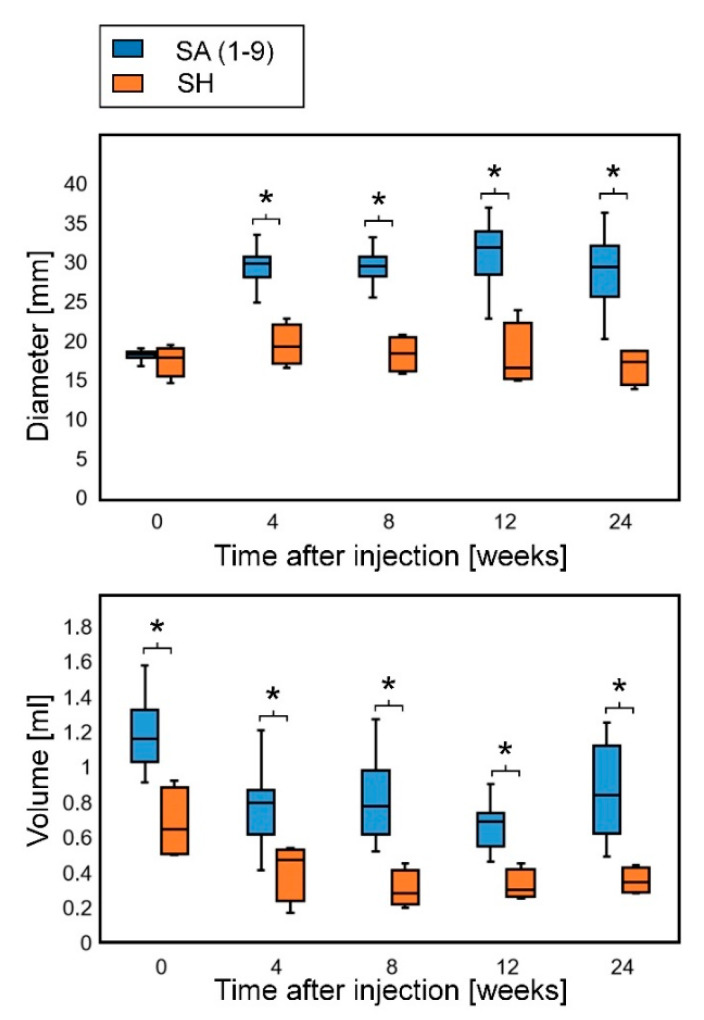
Diameter and volume of subcutaneously injected substances: comparison between SA and SH. Both the diameters and volumes of SA were significantly larger than those of SH at all time points. Diameters at 4 weeks, *p* = 0.00126; diameters at 8 weeks, *p* = 0.00126; diameters at 12 weeks, *p* = 0.00159; diameters at 24 weeks, *p* = 0.00126; volumes at the day of injection, *p* = 0.00147; volumes at 4 weeks, *p* = 0.00769; volumes at 8 weeks, *p* = 0.00181; volumes at 12 weeks, *p* = 0.00288; volumes at 24 weeks, *p* = 0.00189 (Mann–Whitney U-test). *: significant difference (*p* < 0.05). SA: sodium alginate, SH, sodium hyaluronate.

Some rats had a habit of rubbing the crown against the wall, and the shape change of the substance injected on the periosteum varied greatly among individuals. It was difficult to constantly inject 0.1 mL of filler into the thin skin of rats intradermally and examination of the removed specimens revealed that many individuals had the injected substance leaked subcutaneously. Therefore, further evaluation was performed using only the results of subcutaneous injection. When the SA samples were analyzed as one group, SA showed higher volume expansion efficiency than SH, and both the diameters and volumes of SA were significantly larger than those of SH at all time points during the observation period (Figure 2).

**Figure 3:**
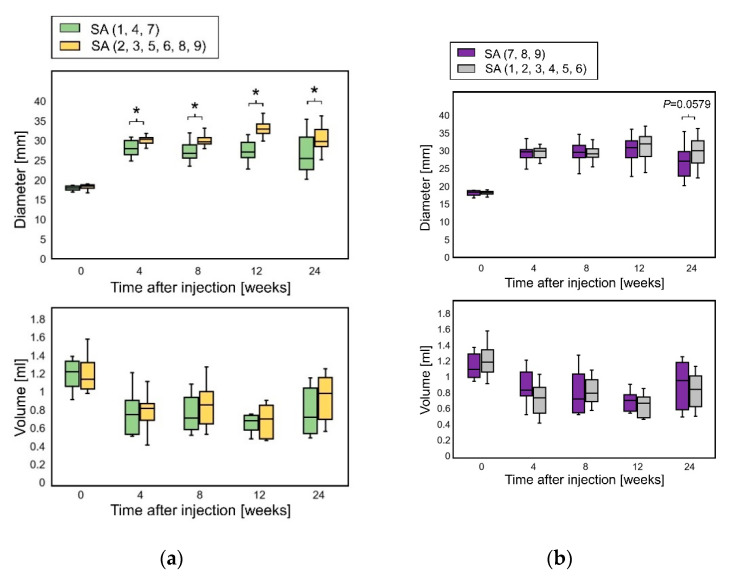
Diameter and volume of subcutaneously injected SA. (**a**) Comparison between SA with a low M/G ratio (SA1, SA4, and SA7) and the other SA types. Diameters at 4 weeks, *p* = 0.00531; diameters at 8 weeks, *p* = 0.00238; diameters at 12 weeks; *p* = 0.00000539; diameters at 24 weeks, *p* = 0.0197 (Mann–Whitney U-test). *, significant difference (*p* < 0.05). SA: sodium alginate, SH, sodium hyaluronate. (**b**) Comparison between SA with high viscosity (SA7, SA8 and SA9) and the other SA types. Diameters at 24 weeks, *p* = 0.0579 (Mann–Whitney U-test). SA: sodium alginate.

Some SA types (SA4, SA7, and SA8) maintained a relatively high elevation of the skin. SA4 and SA7 have a low M/G ratio, and SA7 and SA8 have a high viscosity. SA1 has a low M/G ratio but a low viscosity, and SA9 has a high viscosity and a high M/G ratio. These results suggest that a lower M/G ratio or a higher viscosity of SA is related to higher elevation and volume retention, and the combination of the M/G ratio and viscosity appeared to be influential. Comparing the SA group with a low M/G ratio (SA1, SA4, and SA7) with the others, the subcutaneous diameters of SA with a low M/G ratio at 4 weeks were significantly smaller than those of the others, whereas no significant differences were found in subcutaneous volumes throughout the observation period (Figure 3a). This relationship between the diameters and volumes corroborates that a low M/G ratio contributes to flattening avoidance rather than volume retention. Although no significant differences were found in the subcutaneous diameters and volumes among the high-viscosity SA groups (SA7, SA8, and SA9) throughout the observation period; the subcutaneous diameter of high-viscosity SA at 24 weeks was smaller than that of the others (*p* = 0.0579; Mann–Whitney U-test) (Figure 3b).

### 2.3. Elastica van Gieson Staining

**Figure 4:**
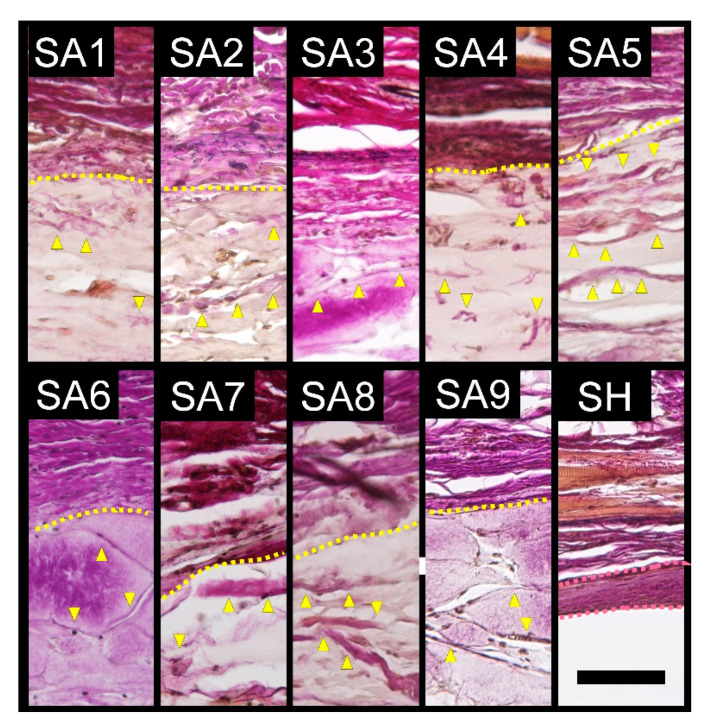
Representative Elastica van Gieson-stained sections at 24 weeks of subcutaneously injected SA or SH. Unlike SH, all the sodium alginate types did not form a capsule around the substance. Instead, collagen fibers were deposited within the substance (yellow arrow heads). The interrupted yellow lines indicate the boundaries between the SA substance and surrounding tissue. The area between the red dotted lines indicate capsule formation surrounding the SH substance. SA: sodium alginate, SH, sodium hyaluronate. Scale bars = 100 µm.

Inflammatory cells infiltrated into the SA-injected area and collagen bands were newly formed inside the area (Figure 4). No capsule formation was observed surrounding the SA-injected area. By contrast, no cells or collagen bands were observed in the injected and surrounding areas of injected SH, but capsule formation around the injected SH was observed. No changes were observed in PBS-injected samples. The average capsule thickness of four rats for each SA and SH is shown in Table 1.

### 2.4. Immunohistochemical Staining of Macrophages

**Figure 5:**
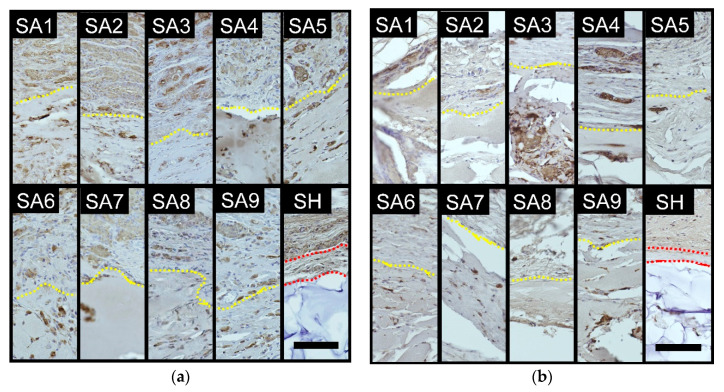
Representative images of immunostained sections for macrophages (Galectin 3). The dotted yellow lines indicate the boundaries between the test substance and surrounding tissue. The area between the red dotted lines indicates capsule formation surrounding SA. SA: sodium alginate, SH, sodium hyaluronate. Scale bars = 100 µm. (**a**) Four weeks after injection. Numerous macrophages aggregated around the SA substance and infiltrated into it, indicating granulomatous reactions. Macrophages also aggregated around the SH substance, but the number was relatively small. (**b**) Twenty-four weeks after injection. The numbers of macrophages were significantly lower (especially in SH) than those at 4 weeks. Some sustained granulomatous reactions were observed in SA1 and SA4.

At 4 weeks, many macrophages were observed around the injected SA in all the groups (Figure 5a). In some cases, the macrophages were highly aggregated (i.e., granulomatous reaction). Granulomatous reactions were particularly strong in the SA1, SA2, SA4, and SA5 groups. Macrophages were also detected around the injected SH, but the number was smaller than that observed in the SA groups, although granulomatous reactions were also observed in some areas around SH.

At 24 weeks, macrophages were observed around the injected substance in all the SA groups, but the number at 24 weeks was generally smaller than at 4 weeks (Figure 5b). Macrophage aggregations were also generally reduced, while granulomatous reactions were still strong in the SA1 and SA4 groups. The number of macrophages around the injected SH was fairly small and the macrophages were hardly aggregated.

The scores of the number of macrophages around the injected substance and the degree of granulomatous reactions are shown in Table 2.

## 3. Discussion

The principal aim of this study was to evaluate the potential of SA as a filler. We compared SA with SH, which is currently the most widely used filler.

Both the volumes of SA and SH increased immediately after injection by absorbing water and then gradually decreased over time. However, the volumes in some of the SA groups increased slightly after 8 weeks. The cause may be due to cell infiltration and the construction of collagen fibers inside the SA substance, as observed in Elastica van Gieson staining. The volume of SH, which had capsule formation and no cell infiltration inside, decreased monotonically throughout the entire experimental period. SH implantation induced capsule formation with low inflammatory cell infiltration, whereas SA caused granulomatous changes, such as the infiltration of inflammatory cells and production of collagen fibers in the injected substance. These differences resulted in higher long-term volume retention of SA with relatively higher inflammation. When compared among sodium alginates, it was observed that those with a low M/G ratio or those with a low viscosity tend to cause a strong inflammatory response in this study, although there has been no previous report regarding property differences by M/G ratio and the underlying mechanism remains unknown. In 2010, Novabel (Marz Pharma, Frankfurt, Germany), a filler comprising cross-linked alginate was launched in the European market. The purified alginate filler was formed into small particles of around 150 micrometers with a three-dimensional structure called “Geleon”. Because Novabel was smooth to inject due to the low viscosity and “Geleon” technology, it was supposed to be ideal to fill hollow eye rings [16]. However, its sale was discontinued because of side effects such as redness, induration, pain, and granuloma formation in the same year. Alginate is considered relatively non-immunogenic, but a mild inflammatory response to alginate was observed in a study using sheep [17]. Additionally, protein contaminants may increase the immunogenicity of alginate [18]. Several reports have demonstrated that the inflammatory reaction at the injection site of Novabel disappears after 4–6 months [19,20]. These clinical courses are consistent with the results of this study. In this experiment using rat models, increased macrophage recruitment and intense granulomatous reactions were observed at 4 weeks; however, in reactions at 24 weeks, fewer macrophages and little or no granulomatous reactions were detected. We previously conducted an SA implantation experiment using pigs (Mexican hairless pig) and obtained similar results in that the inflammatory reaction was strong four weeks after injection and almost disappeared at 24 weeks (data not shown). Filardo et al. reported no inflammatory response in SA implantation experiments using rabbit and sheep models [8]. They obtained samples only 24 weeks after injection. Given that the initial inflammatory findings had subsided by 24 weeks, their results are consistent with our results. When compared among sodium alginates, it was observed that those with a low M/G ratio or those with a low viscosity tend to cause a strong inflammatory response in this study, although there is no previous report regarding property differences by M/G ratio and the underlying mechanism remains unknown.

Despite the failure of Novabel, the occurrence of side effects may be reduced by adjusting the M/G ratio and viscosity. Further research is needed to establish an optimal formulation that can minimize the inflammatory response while maintaining uplift. SA with an ideal viscosity and M/G ratio may become an excellent filler that is relatively low cost, soft, and easy to inject, and which also prevents capsule formation and maintains the volume for a long time.

## 4. Materials and Methods

### 4.1. Test Substances

Nine types of 4% SA phosphate-buffered saline solutions with different M/G ratios or viscosities and commercial sodium hyaluronate (SH) filler (Restylane LIDO; Q-Med, Uppsala, Sweden) as the control substance were used in this study. The nine types of SA solutions were referred to as SA1 to SA9, respectively (Table 3). The M/G ratio ranged from 0.3 to 1.7 and the viscosity ranged from 1.0 ± 0.0 Pa·s to 23.2 ± 0.1 Pa·s. The M/G ratio depends on the type of seaweed used as the raw material and the viscosity were adjusted by heat treatment of enzymatic treatment. The extrusion force, which is required to push out each syringe was measured. We did not feel any resistance when pushing out any type of syringe and all types could be injected smoothly. All of the SA solutions were prepared and supplied by Kaigen Pharma Co., Ltd. (Osaka, Japan).

### 4.2. Animals

Sixty male 8-week-old Wistar rats (Japan SLC, Hamamatsu, Japan) were used in this study. Among them, 20 and 40 rats were used in short-term and long-term experiments, respectively. For each test substance, two and four rats were used in the short-term and long-term experiments, respectively. The study protocol was approved by the animal experiment committee of Jichi Medical University (Issue 17026-01, February 2018).

### 4.3. Injection Procedure

**Figure 6:**
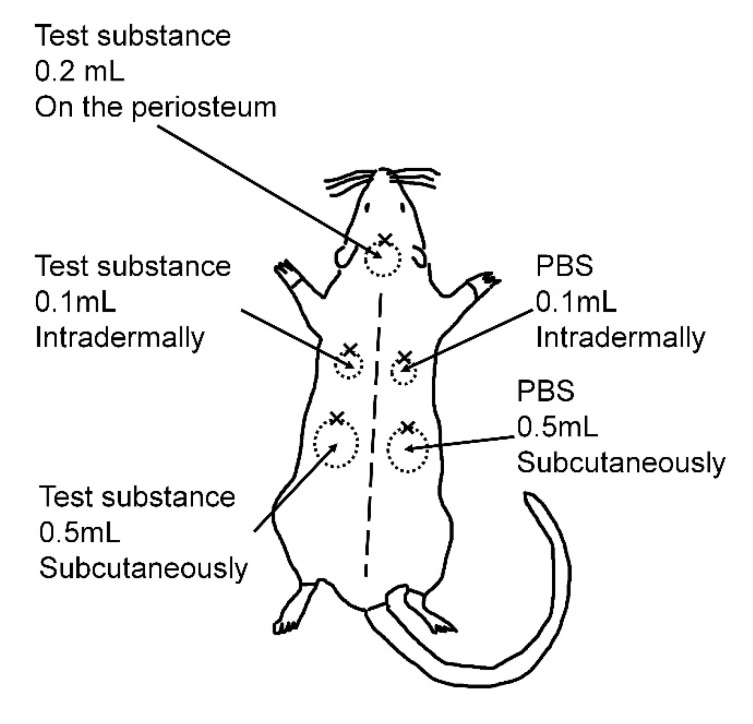
Schema representing injection sites. The test substance (0.2 mL) was injected onto the parietal periosteum, 0.1 mL was injected intradermally in the left scapular area, and 0.5 mL was injected subcutaneously in the left lumbar area. As a negative control, 0.1 mL of phosphate-buffered saline (PBS) was injected intradermally in the right scapular area and 0.5 mL was injected subcutaneously in the right lumbar area. A 6-0 nylon thread was ligated to the cephalic side of each injection site as a landmark.

The injections were performed under general anesthesia with isoflurane inhalation. The hairs on the back were shaved using hair clippers, followed by hair removal with a depilatory cream (Epilat; Kracie Pharma, Tokyo, Japan). Each test substance was injected onto the parietal periosteum (0.2 mL), intradermally in the left scapular area (0.1 mL), and subcutaneously in the left lumbar area (0.5 mL) (Figure 6). As a negative control, the same volumes of phosphate-buffered saline (PBS) were injected on the right side in the same manner. A 1-mL Luer-lock syringe (TOP Corporation, Tokyo, Japan) with a 30-gauge needle (NIPRO Corporation, Osaka, Japan) was used to inject the test substance. A 6-0 nylon thread was used to make a landmark of each injection site. The rats were maintained under a 12-h day/night cycle and were fed standard feed with water ad libitum. At the end of each experimental period (4 weeks, short-term experiment; 24 weeks, long-term experiment), the rats were euthanized by anesthetic overdose. Assuming clinical use, the injections were given to three different layers and the injection volumes were determined as the minimum amount, with which the swelling could be confirmed at each layer: 0.2 mL on the periosteum, 0.1 mL intradermally, and 0.5 mL subcutaneously.

### 4.4. Diameter and Volume Measurements

In the long-term experiment, the diameter of the skin bulges due to the injected substance was measured using a caliper and the volume of the residual substance was measured using a micro X-ray computed tomography (CT) system (Latheta LCT-200; Hitachi, Tokyo, Japan) at each time point (injection day, 4 weeks, 8 weeks, 12 weeks, and 24 weeks after injection) (Figure S1). The pixel size was set at 240 µm, and the thickness and interval of the slices were set at 240 µm and 1260 µm, respectively. The area of the injected substance was determined in each slice and then was multiplied by the slice interval (1.26 mm); the products were summed to determine the substance volume. Photoshop CC (Adobe Systems Incorporated, San Jose, CA, USA) was used for the calculation.

### 4.5. Histopathological Analysis

After euthanization, the tissues of the injected site were excised using a scalpel, fixed in 4% paraformaldehyde phosphate buffer solution (FUJIFILM Wako Pure Chemical Corporation, Osaka, Japan) at 4 °C for 24 h, embedded in paraffin, sectioned at 6 µm and mounted on microslides. The samples were subjected to Hematoxylin and Eosin staining and several other staining types described below.

### 4.6. Elastica van Gieson Staining

All the samples of the long-term experiment were subjected to Elastica van Gieson staining to observe capsule formation around the injected substances. They were treated with 1% hydrochloric acid alcohol (Muto Pure Chemicals, Tokyo, Japan) and immersed in resorcin–fuchsin solution (Muto Pure Chemicals) for 60 min. After rinsing in tap water for 5 min, the samples were stained with Weigert’s iron hematoxylin stain solution (Muto Pure Chemicals) for 5 min. The samples were then treated with 1% hydrochloric acid alcohol (Muto Pure Chemicals) and rinsed in tap water for 10 min. Finally, the samples were stained with van Gieson solution (Muto Pure Chemicals) for 5 min. The thickness of the capsule around the subcutaneously injected SA or SH was measured at randomly selected three locations for each rat by three evaluators (M.M., T.M., and Y.W) in a blind form and the average was calculated. Photoshop CC (Adobe Systems Incorporated) was used for the measurement.

### 4.7. Immunohistochemical Staining of Macrophages

To observe the inflammatory response to the injected substances, immunohistochemical staining of macrophages was performed using samples in which the test substances were injected subcutaneously. For antigen unmasking, the samples were boiled in pH 6.0, 0.01 mol/L citrate buffer solution (LSI Medience Corporation, Tokyo, Japan) for 15 min. After washing in 0.01 mol/L of PBS (FUJIFILM Wako Pure Chemical Corporation), the samples were immersed twice in polyoxyethylene octylphenyl ether solution (FUJIFILM Wako Pure Chemical Corporation) for 5 min for permeabilization. To quench endogenous peroxidase activity, the samples were immersed in 0.3% hydrogen peroxide solution (FUJIFILM Wako Pure Chemical Corporation) for 30 min and washed in 0.01 mol/L of PBS for 5 min. After incubation with 2.5% normal horse serum (Vector Laboratories, Burlingame, CA, USA) for 20 min, the samples were incubated with a 1:500 dilution of rabbit-derived anti-Galectin 3 primary antibody (GeneTex, Irvine, CA, USA) at 4 °C overnight. After washing in 0.01 mol/L of PBS for 5 min, the samples were incubated with diluted solution of horse-derived anti-rabbit IgG secondary antibody with enzyme micropolymer (ImmPRESS Reagent; Vector Laboratories) at room temperature for 30 min. After washing in 0.01 mol/L of PBS for 5 min, the samples were incubated in peroxidase substrate solution (ImmPACT DAB; Vector Laboratories) for 20 s for color development, followed by rinsing in tap water for 5 min. Finally, the samples were immersed in Carrazzi’s hematoxylin solution (Muto Pure Chemicals) for 5 s for nuclear staining and rinsed in tap water for 5 min.

The number of macrophages around the injected substance and the degree of granulomatous reactions were scored in three and four grades, respectively by three blinded evaluators (M.M., T.M., and Y.W). The number of macrophages contained in randomly selected 5 fields was counted and the degree was determined by the average number (++, *n* > 100 cells; +, 100 ≥ *n* >10 cells; ±, *n* ≤ 10 cells). The degree of granulomatous reactions was visually scored from Hematoxylin and Eosin stained specimens in four grades (3, severe; 2, moderate; 1, mild; 0, absent) in accordance with a modification of the scoring system described by Santos et al. [21] using a visual four-grade scoring criteria shown in Figure S2.

### 4.8. Statistical Analysis

The diameters and volumes of SA and SH were analyzed using the Mann–Whitney U-test. All *p*-values were two sided, and *p* ≤ 0.05 were considered statistically significant. All statistical analyses were performed using a software named “EZR” (Saitama Medical Center, Jichi Medical University, Saitama, Japan), which is a graphical user interface for R (The R Foundation for Statistical Computing, Vienna, Austria). More precisely, it is a modified version of R commander designed to add statistical functions and is frequently used in biostatistics [22].

## Figures and Tables

**Table 1:** The capsule thickness of four rats for each SA and SH. The data are shown as the mean ± standard. SA: sodium alginate, SH: sodium hyaluronate.

The Capsule Thickness (µm)	
SA1	0.0 ± 0.0
SA2	0.0 ± 0.0
SA3	0.0 ± 0.0
SA4	0.0 ± 0.0
SA5	0.0 ± 0.0
SA6	0.0 ± 0.0
SA7	0.0 ± 0.0
SA8	0.0 ± 0.0
SA9	0.0 ± 0.0
SH	52.9 ± 7.2

**Table 2:** The scores of the number of macrophages and the degree of granulomatous reactions. For the degree of granulomatous reactions, the average of the scores of the three evaluators is shown: ++, 3 ≥ *n* > 2; +, 2 ≥ *n* > 1; ±, 1 ≥ *n* >0; –, 0. SA: sodium alginate, SH: sodium hyaluronate.

Macrophages/Granulomatous Reaction		
	4 Weeks	24 Weeks
SA1	++/++	+/++
SA2	++/++	+/+
SA3	++/+	+/±
SA4	++/++	+/++
SA5	++/++	+/+
SA6	++/+	+/+
SA7	++/+	+/±
SA8	++/+	+/–
SA9	++/+	+/±
SH	+/–	±/–

**Table 3:** Properties of the tested sodium alginate and commercial hyaluronic fillers. The data are shown as the mean ± standard deviation of triplicates. There is no M/G ratio for sodium hyaluronate. SA: sodium alginate, SH: sodium hyaluronate.

Type	M/G Ratio †	Viscosity (Pa·s)	Extrusion Force (N)
SA1	0.3	1.0 ± 0.0	10.9 ± 0.0
SA2	1.4	1.3 ± 0.0	14.7 ± 0.0
SA3	1.7	1.5 ± 0.0	15.0 ± 0.0
SA4	0.3	10.1 ± 0.1	15.8 ± 0.1
SA5	1.1	8.4 ± 0.0	14.9 ± 0.0
SA6	1.6	8.5 ± 0.4	16.4 ± 0.4
SA7	0.4	18.2 ± 0.2	16.0 ± 0.2
SA8	1.4	19.9 ± 0.1	16.8 ± 0.1
SA9	1.6	15.8 ± 0.1	17.1 ± 0.1
SH	N/A	23.2 ± 0.1	11.1 ± 0.1

† Ratio of mannuronate and guluronate.

## Supplementary Materials

The following are available online at https://www.mdpi.com/1660-3397/18/10/520/s1, Figure S1: Representative images of a micro X-ray computed tomography system immediately after injection of sodium alginate, Table S1: Sequential changes in the diameter, Table S2: Sequential changes in the volume.
