# Supplementary material for: Sodium Alginate as a Potential Therapeutic Filler: An In Vivo Study in Rats
Source: Mar Drugs. 2020 Oct 19;18(10):520. doi: 10.3390/md18100520 (PMC7589138; doi:10.3390/md18100520)
Supplement: Supplementary file 1 [file marinedrugs-18-00520-s001.pdf]

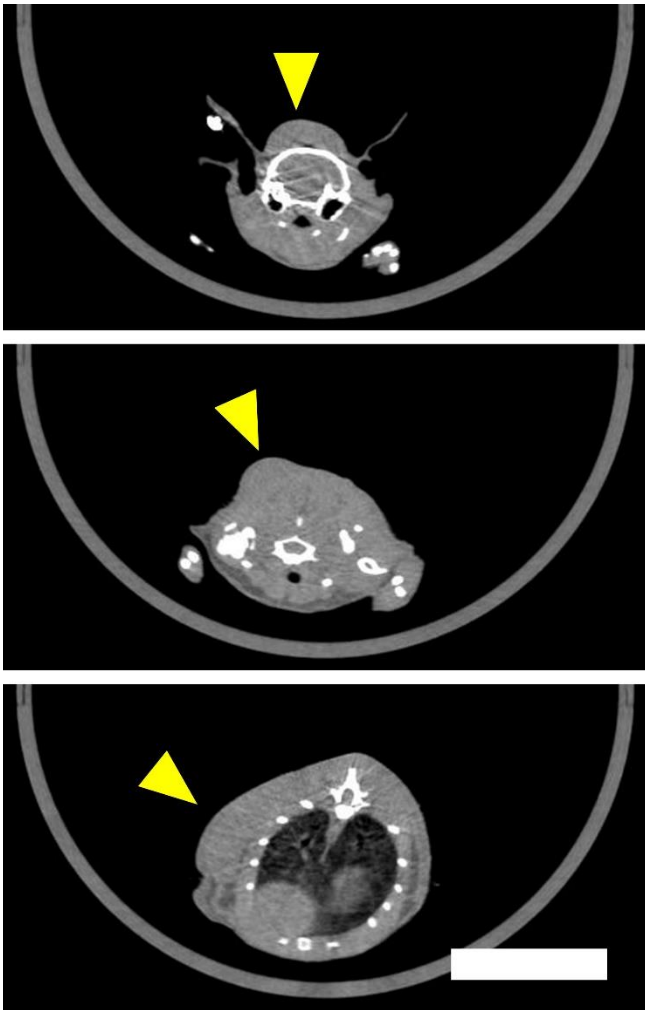

Figure S1. Representative images of a micro X-ray computed tomography system immediately after injection of sodium alginate. (Above) 0.2 mL injected onto the parietal periosteum. (Middle) 0.1 mL injected intradermally. (Below) 0.5 mL injected subcutaneously. Scale bars = 30 mm.

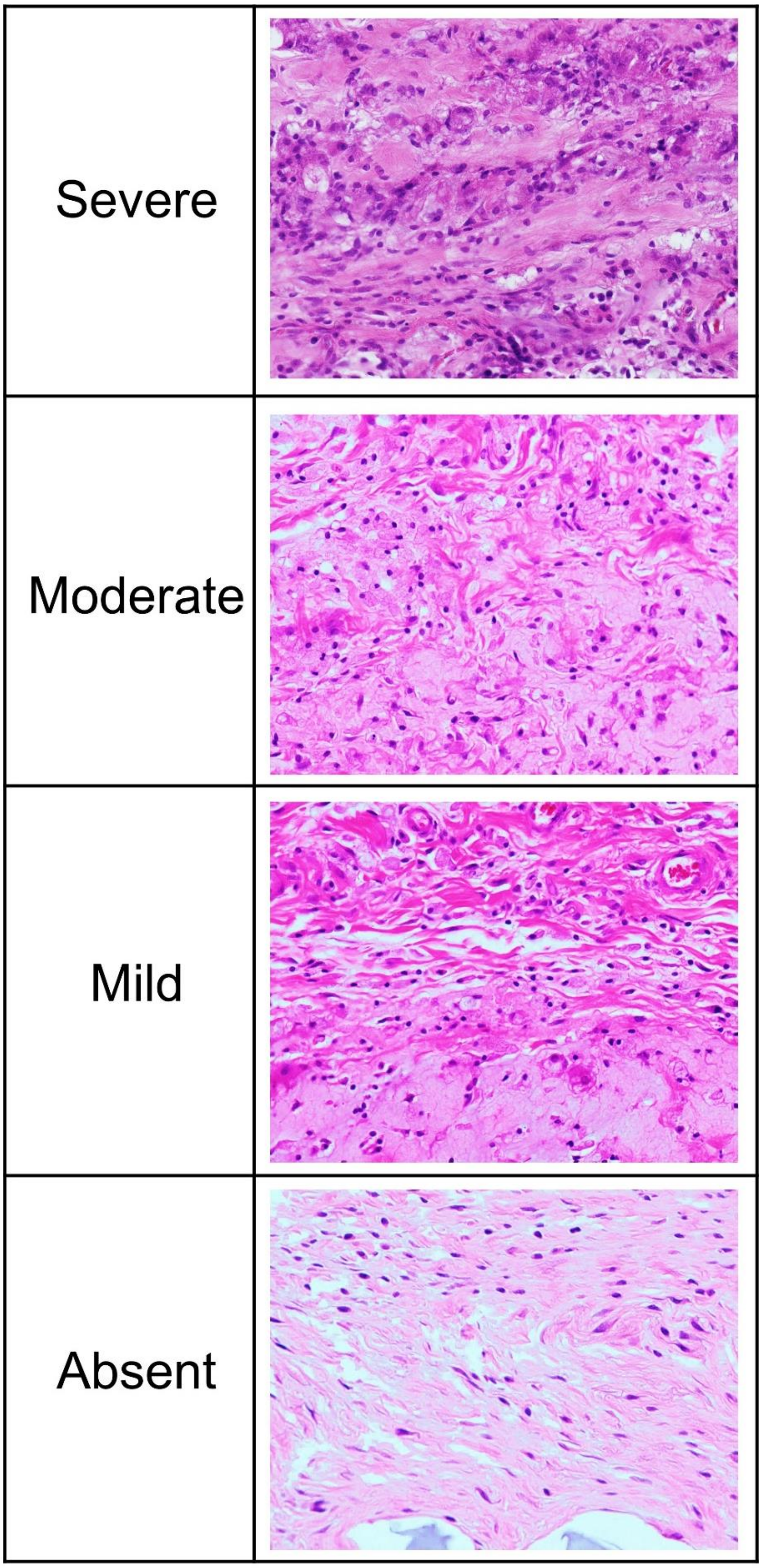

Figure S2. Visual scoring scale for granulomatous changes

Table S1. The results are shown as means ± standard error. The numbers are rounded off to the first decimal place. Four rats for each test substance (n = 4). Some bulges were flattened, and their diameters could not be measured (*n = 3; †n = 1; N/A, n = 0). SA: sodium alginate, SH: sodium hyaluronate.

Diameter [mm]

		Day 0	4 weeks	8 weeks	12 weeks	24 weeks
	Onto the periosteum	12.3 ± 0.3	15.8 ± 1.6	16.8 ± 1.7	15.2†	N/A
SA1	Intradermally	10.8 ± 0.5	13.9 ± 0.6	13.8 ± 0.9	14.4 ± 1.7	13.4 ± 0.9
	Subcutaneously	17.7 ± 0.3	29.6 ± 0.7	27.0 ± 0.7	28.8 ± 1.3	29.2 ± 1.6
	Onto the periosteum	12.5 ± 0.2	16.6 ± 0.8	16.5 ± 1.4	14.9†	N/A
SA2	Intradermally	10.4 ± 0.4	14.3 ± 0.5	13.9 ± 1.1	15.4 ± 1.0	16.3 ± 0.3
	Subcutaneously	18.3 ± 0.1	29.7 ± 1.0	30.1 ± 0.7	31.8 ± 0.8	33.1± 1.2
	Onto the periosteum	12.7 ± 0.2	14.8 ± 0.8	14.2 ± 0.8	17.0†	N/A
SA3	Intradermally	11.3 ± 0.2	13.7 ± 0.4	15.7 ± 1.2	14.5 ± 1.4*	14.1± 0.7*
	Subcutaneously	18.6 ± 0.1	29.9 ± 0.4	29.5 ± 0.7	34.6 ± 1.1	30.9 ± 1.1
	Onto the periosteum	11.6 ± 0.5	17.8 ± 0.9	16.7 ± 0.4	15.4 ± 0.5	14.9 ± 0.9
SA4	Intradermally	10.8 ± 0.2	13.7 ± 0.3	12.9 ± 0.7	13.4 ± 1.0	12.0 ± 0.9
	Subcutaneously	18.2 ± 0.3	26.9 ± 0.4	27.0 ± 0.8	26.0 ± 0.8	24.7 ± 1.1
	Onto the periosteum	12.5 ± 0.1	17.1 ± 1.1	14.8 ± 0.7	15.1 ± 0.5*	11.3†
SA5	Intradermally	10.9 ± 0.3	14.5 ± 0.5	15.8 ± 0.7	16.2 ± 1.4	16.1 ± 0.6
	Subcutaneously	18.1 ± 0.4	30.6 ± 0.3	31.6 ± 0.8	34.2 ± 1.0	30.1 ± 1.8
	Onto the periosteum	12.5 ± 0.3	16.8 ± 0.5	15.0 ± 0.6	N/A	N/A
SA6	Intradermally	10.4 ± 0.5	14.8 ± 0.4	16.1 ± 1.0	16.0 ± 0.5	16.6 ± 0.9
	Subcutaneously	18.0 ± 0.4	30.0 ± 0.7	29.4 ± 0.4	33.2 ± 0.6	30.8 ± 1.8
	Onto the periosteum	12.6 ± 0.1	18.4 ± 1.0	17.8 ± 0.7	15.9 ± 1.2	14.1 ± 0.8*
SA7	Intradermally	10.3 ± 0.3	13.4 ± 0.5	12.9 ± 0.5	13.0 ± 0.6	11.6 ± 0.8
	Subcutaneously	18.0 ± 0.4	27.5 ± 1.4	28.2 ± 2.1	26.5 ± 1.5	25.8 ± 3.4
	Onto the periosteum	12.8 ± 0.4	18.6 ± 0.5	18.2 ± 0.7	16.2 ± 0.9	16.3 ± 0.7*
SA8	Intradermally	10.9 ± 0.2	14.3 ± 0.5	14.7 ± 0.9	14.7 ± 1.1	13.6 ± 0.8
	Subcutaneously	18.0 ± 0.5	29.0 ± 0.5	29.4 ± 0.6	31.5 ± 0.7	26.1 ± 2.0
	Onto the periosteum	12.2 ± 0.2	17.1 ± 1.1	17.1 ± 0.6	13.4†	N/A
SA9	Intradermally	10.8 ± 0.2	15.8 ± 0.5	16.8 ± 1.4	18.3 ± 1.0	15.3 ± 1.0
	Subcutaneously	18.3 ± 0.4	31.7 ± 0.9	30.6 ± 1.4	33.5 ± 1.1	28.7 ± 1.1
	Onto the periosteum	12.4 ± 0.4	14.2 ± 0.6	13.6 ± 0.8	12.1 ± 0.4	10.0 ± 0.2
SH	Intradermally	10.6 ± 0.6	11.4 ± 0.9	9.9 ± 0.6	8.9 ± 0.5	7.6 ± 0.5
	Subcutaneously	17.4 ± 1.0	19.4 ± 1.3	18.3 ± 1.1	17.9 ± 2.0	16.8 ± 1.2

Table S2. The results are shown as means ± standard error. The numbers are rounded off to the second decimal place. Four rats for each test substance (n = 4). Some boundaries of the injected substances were unclear in the CT images, and their volumes could not be measured (*n = 3; ⁑ n = 2; †n = 1; N/A, n = 0). SA: sodium alginate, SH: sodium hyaluronate.

Volume [mL]

		**Day 0**	**4 weeks**	**8 weeks**	**12 weeks**	**24 weeks**
A1	Onto the periosteum	0.52 ± 0.03	N/A	N/A	N/A	0.25†
	Intradermally	0.25 ± 0.01	0.17 ± 0.02 ⁑	0.10†	0.12†	N/A
	Subcutaneously	1.23 ± 0.08	0.53 ± 0.01*	0.72 ± 0.11 ⁑	0.62 ± 0.14 ⁑	0.54 ± 0.04 ⁑
A2	Onto the periosteum	0.56 ± 0.03	N/A	N/A	N/A	N/A
	Intradermally	0.25 ± 0.01	N/A	N/A	N/A	N/A
	Subcutaneously	1.23 ± 0.05	0.41†	0.80 ± 0.11*	N/A	0.73 ± 0.11 ⁑
A3	Onto the periosteum	0.55 ± 0.02	N/A	N/A	N/A	N/A
	Intradermally	0.25 ± 0.02	N/A	N/A	N/A	N/A
	Subcutaneously	1.22 ± 0.09	0.67†	0.71†	0.46†	0.76 ± 0.05 ⁑
A4	Onto the periosteum	0.51 ± 0.03	0.47 ± 0.08*	0.33 ± 0.09*	0.45 ± 0.02 ⁑	0.27 ± 0.10 ⁑
	Intradermally	0.26 ± 0.01	0.14 ± 0.03	0.17 ± 0.04	0.14 ± 0.02	0.08 ⁑
	Subcutaneously	1.17 ± 0.09	0.87 ± 0.06	0.85 ± 0.09	0.66 ± 0.03	0.93 ± 0.09
A5	Onto the periosteum	0.53 ± 0.04	N/A	N/A	N/A	N/A
	Intradermally	0.26 ± 0.01	0.16 ± 0.01	0.16 ± 0.03 ⁑	0.10†	N/A
	Subcutaneously	1.29 ± 0.14	0.76 ± 0.12 ⁑	0.96 ± 0.02*	0.67 ± 0.19 ⁑	0.89 ± 0.17*
A6	Onto the periosteum	0.58 ± 0.03	N/A	N/A	N/A	N/A
	Intradermally	0.25± 0.01	0.11*	N/A	N/A	N/A
	Subcutaneously	1.14 ± 0.08	0.80 ± 0.07 ⁑	0.57†	0.73†	1.01 ± 0.03 ⁑
A7	Onto the periosteum	0.63 ± 0.01	0.45 ± 0.03 ⁑	0.42 ± 0.02 ⁑	0.44 ± 0.02 ⁑	0.30 ± 0.02 ⁑
	Intradermally	0.21 ± 0.01	0.11 ± 0.01*	0.10 ± 0.01	0.20 ± 0.01 ⁑	0.16 ± 0.06 ⁑
	Subcutaneously	1.17 ± 0.10	0.82 ± 0.20*	0.67 ± 0.12	0.67 ± 0.05*	0.74 ± 0.15
A8	Onto the periosteum	0.51 ± 0.02	0.47 ± 0.05 ⁑	0.36 ⁑	0.38 ± 0.08 ⁑	0.17†
	Intradermally	0.24 ± 0.02	0.22 ± 0.05	0.15 ± 0.03	0.18 ± 0.02*	0.19†
	Subcutaneously	1.05 ± 0.04	0.94 ± 0.07	0.92 ± 0.16	0.71 ± 0.10*	1.20 ± 0.02*
A9	Onto the periosteum	0.55 ± 0.05	N/A	0.46†	N/A	N/A
	Intradermally	0.26 ± 0.01	0.20†	0.25†	N/A	N/A
	Subcutaneously	1.16 ± 0.08	0.81 ± 0.02 ⁑	0.79 ± 0.14 ⁑	N/A	0.68†
H	Onto the periosteum	0.22 ± 0.01	0.18 ± 0.03*	0.20 ± 0.01*	0.18 ± 0.03*	0.13 ± 0.01
	Intradermally	0.12 ± 0.01	0.11 ± 0.01	0.08 ± 0.01	0.08 ± 0.02	0.12 ± 0.01
	Subcutaneously	0.68 ± 0.10	0.41 ± 0.08	0.30 ± 0.05	0.33 ± 0.04	0.35 ± 0.04
